# Effects and moderators of psychosocial interventions on quality of life, and emotional and social function in patients with cancer: An individual patient data meta‐analysis of 22 RCTs

**DOI:** 10.1002/pon.4648

**Published:** 2018-03-15

**Authors:** J. Kalter, I.M. Verdonck‐de Leeuw, M.G. Sweegers, N.K. Aaronson, P.B. Jacobsen, R.U. Newton, K.S. Courneya, J.F. Aitken, J. Armes, C. Arving, L.J. Boersma, A.M.J. Braamse, Y. Brandberg, S.K. Chambers, J. Dekker, K. Ell, R.J. Ferguson, M.F.M. Gielissen, B. Glimelius, M.M. Goedendorp, K.D. Graves, S.P. Heiney, R. Horne, M.S. Hunter, B. Johansson, M.L. Kimman, H. Knoop, K. Meneses, L.L. Northouse, H.S. Oldenburg, J.B. Prins, J. Savard, M. van Beurden, S.W. van den Berg, J. Brug, L.M. Buffart

**Affiliations:** ^1^ Department of Epidemiology and Biostatistics, Amsterdam Public Health Research Institute VU University Medical Center Amsterdam The Netherlands; ^2^ Department of Clinical Psychology VU University Amsterdam The Netherlands; ^3^ Department of Otolaryngology‐Head and Neck Surgery, Amsterdam Public Health research institute and Cancer Center Amsterdam VU University Medical Center Amsterdam The Netherlands; ^4^ Division of Psychosocial Research and Epidemiology Netherlands Cancer Institute Amsterdam The Netherlands; ^5^ Division of Cancer Control and Population Science National Cancer Institute Bethesda, Maryland FL USA; ^6^ Exercise Medicine Research Institute Edith Cowan University Joondalup WA Australia; ^7^ Faculty of Kinesiology, Sport, and Recreation University of Alberta Edmonton AB Canada; ^8^ Menzies Health Institute Queensland Griffith University Southport Australia; ^9^ Cancer Council Queensland Brisbane Australia; ^10^ Institute for Resilient Regions University of Southern Queensland Brisbane Australia; ^11^ Faculty of Health and Medical Sciences University of Surrey Guildford UK; ^12^ Department of Immunology, Genetics and Pathology Uppsala University Uppsala Sweden; ^13^ Department of Radiation Oncology Maastricht University Medical Center (MAASTRO clinic) Maastricht The Netherlands; ^14^ GROW―School for Oncology and Developmental Biology Maastricht University Medical Center Maastricht The Netherlands; ^15^ Department of Medical Psychology Academic Medical Center Amsterdam The Netherlands; ^16^ Department of Oncology‐Pathology Karolinska Institute Stockholm Sweden; ^17^ Prostate Cancer Foundation of Australia Sydney NSW Australia; ^18^ Department of Rehabilitation Medicine VU University Medical Center Amsterdam The Netherlands; ^19^ Department of Psychiatry VU University Medical Center Amsterdam The Netherlands; ^20^ Department of Adults and Healthy Aging University of Southern California Los Angeles CA USA; ^21^ Division of Hematology‐Oncology University of Pittsburgh Cancer Institute Pittsburgh PA USA; ^22^ Department of Health Psychology University Medical Center Groningen, University of Groningen Groningen The Netherlands; ^23^ Lombardi Comprehensive Cancer Center Georgetown University Washington, DC USA; ^24^ College of Nursing University of South Carolina Columbia SC USA; ^25^ UCL School of Pharmacy University College London London UK; ^26^ Institute of Psychiatry, Psychology and Neuroscience King's College London London UK; ^27^ Department of Clinical Epidemiology and Medical Technology Assessment Maastricht University Medical Centre Maastricht The Netherlands; ^28^ University of Alabama at Birmingham School of Nursing Birmingham AL USA; ^29^ University of Michigan School of Nursing Ann Arbor MI USA; ^30^ Department of Surgical Oncology Netherlands Cancer Institute/Antoni van Leeuwenhoek Hospital Amsterdam The Netherlands; ^31^ Department of Medical Psychology Radboud University Medical Center Nijmegen The Netherlands; ^32^ School of Psychology Université Laval and Laval University Cancer Research Center Québec QC Canada; ^33^ Department of Gynecology Netherlands Cancer Institute/Antoni van Leeuwenhoek Hospital Amsterdam The Netherlands; ^34^ Amsterdam School of Communication Research (ASCoR) University of Amsterdam Amsterdam The Netherlands; ^35^ Department of Medical Oncology, Cancer Center Amsterdam VU University Medical Center Amsterdam The Netherlands

**Keywords:** coping skills training, individual patient data meta‐analysis, neoplasm, psychosocial care, psychotherapy, quality of life

## Abstract

**Objective:**

This individual patient data (IPD) meta‐analysis aimed to evaluate the effects of psychosocial interventions (PSI) on quality of life (QoL), emotional function (EF), and social function (SF) in patients with cancer, and to study moderator effects of demographic, clinical, personal, and intervention‐related characteristics.

**Methods:**

Relevant studies were identified via literature searches in 4 databases. We pooled IPD from 22 (n = 4217) of 61 eligible randomized controlled trials. Linear mixed‐effect model analyses were used to study intervention effects on the post‐intervention values of QoL, EF, and SF (z‐scores), adjusting for baseline values, age, and cancer type. We studied moderator effects by testing interactions with the intervention for demographic, clinical, personal, and intervention‐related characteristics, and conducted subsequent stratified analyses for significant moderator variables.Results: PSI significantly improved QoL (β = 0.14,95%CI = 0.06;0.21), EF (β = 0.13,95%CI = 0.05;0.20), and SF (β = 0.10,95%CI = 0.03;0.18). Significant differences in effects of different types of PSI were found, with largest effects of psychotherapy. The effects of coping skills training were moderated by age, treatment type, and targeted interventions. Effects of psychotherapy on EF may be moderated by cancer type, but these analyses were based on 2 randomized controlled trials with small sample sizes of some cancer types.

**Conclusions:**

PSI significantly improved QoL, EF, and SF, with small overall effects. However, the effects differed by several demographic, clinical, personal, and intervention‐related characteristics. Our study highlights the beneficial effects of coping skills training in patients treated with chemotherapy, the importance of targeted interventions, and the need of developing interventions tailored to the specific needs of elderly patients.

## 1 BACKGROUND

Previous systematic reviews and meta‐analyses from randomized controlled trials (RCTs) have reported that psychosocial interventions (PSI) significantly reduce psychosocial problems and improve the quality of life (QoL), emotional function (EF), and social function (SF) of patients during and after cancer treatment, but effects sizes vary.^1^, ^2^, ^3^, ^4^, ^5^, ^6^, ^7^, ^8^, ^9^, ^10^, ^11^, ^12^, ^13^ Better insight into intervention moderators can facilitate identifying and subsequently targeting subgroups of patients with cancer that respond best to a particular type of PSI, thereby improving the intervention effects.^14^

Results from individual RCTs have suggested that younger age, female gender, lower socio‐economic status, having breast cancer compared with lung cancer, cancer recurrence, lower self‐esteem, higher depressive symptoms, and lower self‐efficacy moderate the effects of PSI in patients with cancer.^15^, ^16^, ^17^, ^18^, ^19^ However, these findings from individual RCTs should be interpreted with caution as they are generally not designed and powered to study moderators of intervention effects.^20^

Additionally, meta‐analyses on aggregate (summary) data from RCTs have shown that the effects of PSI on psychological well‐being were larger in patients with older age, male gender, lower income, and other types of cancer compared with breast cancer.^6^ Larger effects have also been reported for patients with higher distress and lower QoL at baseline, and who attended a psychotherapeutic or psycho‐educational intervention compared with an information‐only intervention.^1^, ^2^, ^4^, ^5^, ^7^, ^12^ However, a meta‐analysis of summary data relies on mean patient characteristics (eg, the mean age of patients or the proportion of women in a study), which does not allow testing of interactions between the intervention and patient‐level characteristics.^20^ The use of summary data thereby increases the risk for ecological bias, which refers to the failure of associations at the study‐level to correctly reflect associations at the patient‐level caused by confounding factors across trials.^21^ Moderator effects found in aggregate data meta‐analyses should therefore be interpreted with caution.

A meta‐analysis of individual patient data (IPD) involves obtaining and then synthesizing the raw IPD from multiple related studies,^22^ and has the advantage to test interactions between interventions and patient‐level characteristics using the large number of raw data points, conducting subsequent stratified analyses, and standardized analytic techniques across the included studies.^23^, ^24^

The current IPD meta‐analysis is part of the Predicting OptimaL cAncer RehabIlitation and Supportive care (POLARIS) study.^25^ The aims were to evaluate the effects of PSI on QoL, EF, and SF in patients with cancer, and to identify for the first time demographic, clinical, personal, and intervention‐related moderators of intervention effects with IPD meta‐analysis.

## 2 METHODS

### 2.1 Identification and inclusion of studies

Detailed descriptions of the design, procedures, and search strategies of the POLARIS study have been published previously.^25^ Briefly, relevant published and unpublished studies (eg, study protocol papers) were identified via systematic searches in 4 electronic databases (PubMed, EMBASE, PsycINFO, and CINAHL), reference checking of systematic reviews, meta‐analyses, and personal communication with collaborators, colleagues, and other experts in the field.^25^ The original search was conducted in September 2012.^25^ In case an identified study was not yet published, we maintained contact about the study completion date, to allow inclusion at a later stage during the data collection process of approximately 3 years. POLARIS included RCTs that evaluated the effects of physical activity interventions and/or PSI on QoL compared with a wait‐list, usual care, or attention control group in adult patients with cancer.^25^ The effects of physical activity interventions on QoL and physical function have been reported elsewhere.^26^

We used Cunningham's hierarchic classification to distinguish 5 types of heterogenetic PSI, based on the degree of psychological change that the different interventions aim to promote in patients with cancer: (1) information provision, ie, interventions aiming to increase a patient's knowledge of cancer and/or its treatments, side effects, and consequences; (2) support, ie, interventions intended to help patients to cope with the implications of cancer and its treatment, eg, express associated emotions, diminish a sense of isolation, identify unmet needs, take some control over events, deal with family members and health care personnel, and accept losses and changed roles; (3) coping skills training (CST), ie, interventions targeted at attaining new cognitive‐behavioral skills such as relaxation, mental imaging, thought and affect management, and activity planning; (4) psychotherapy, ie, interventions delivered by an appropriately trained professional which aim to achieve a more fundamental psychological change to increase self‐understanding via, for example, psychodynamic therapy, and supportive‐therapeutic approaches; and (5) spiritual or existential therapy, ie, interventions promoting experiential awareness of a transcendent order or power, some sense of belonging to a meaningful universe including mediation and prayer (where meaningful to the patient), appropriate reading, discussion, and reflection around spiritual topics.^27^

For the current IPD meta‐analysis, RCTs on PSI that fit in the first 4 categories were included. Although we acknowledge the potential importance of the fifth category, we excluded RCTs focusing on PSI in this category, because of the heterogeneity of RCTs on PSI in this category (eg, spiritual or existential therapy, including meditation and mindfulness). At this point, we also excluded interventions such as yoga and pain management, as well as diet or multimodal lifestyle interventions (for example physical activity and diet combined), to reduce heterogeneity, and to keep the number of datasets to be retrieved manageable. Based on the description of the intervention provided in the original studies, 2 authors (JK + IVdL) independently classified the type of intervention. Disagreements (9%) were resolved by discussion. All PIs of original studies approved the categorization. The study protocol was registered in PROSPERO in February 2013 (CRD42013003805).^25^

A letter of invitation to join the POLARIS consortium and share data was sent to the principal investigator (PI) of eligible RCTs. In case of no response, we sent reminders or contacted another PI on the same study. After PIs expressed interest in data sharing, they were requested to sign a data sharing agreement stating that they agreed with the POLARIS policy document and were willing to share anonymized data of study participants who were randomized. The data could be supplied in various formats and were checked for completeness, improbable values, consistency with published articles, and missing items. Subsequently, data sets were imported in the POLARIS database where they were re‐coded according to standardized protocols and harmonized.^25^

### 2.2 Representativeness of included studies

To examine whether the included RCTs were a representative sample of all eligible RCTs, we compared pooled effect sizes of RCTs included with those not included. For this purpose, we updated the original search in October 2017 to also include studies that were published recently. Effect sizes per RCT were calculated by subtracting the published average post‐intervention value of QoL, EF, or SF of the control group from that of the intervention group and dividing the result by the pooled standard deviation. We adjusted effect sizes for small samples as suggested by Hedges and Olkin.^28^ Effect sizes (Hedges'g) were pooled with a random effects model and differences in effects between studies providing data and those that did not were examined using Comprehensive Meta‐analysis software (version 2.2.064).

We evaluated publication bias for all eligible studies and for studies providing data by inspecting the funnel plot and by the Duval and Tweedie's trim and fill procedure.^29^, ^30^ The procedure provides estimates of the number of missing studies and the effect size after the publication bias has been taken into account. The Egger's test was used to test whether the bias captured by the funnel plot was significant.

### 2.3 Data extraction and quality assessment of included studies

Two independent researchers (JK + MS) extracted study characteristics and rated the quality of included studies from the published papers. We used the recommended “risk of bias” assessment tool of the Cochrane Collaboration^31^ to grade the quality as high (“+”), low (“−”), or unclear (?) on the following aspects: random sequence generation (high quality if a random assignment was used), allocation concealment (high quality in case of central, computerized allocation or sequentially numbered sealed envelopes), incomplete outcome (high quality if intention‐to‐treat analyses were performed, and less than 10% of the outcome data were missing or adequate imputation techniques were used), and incomplete reporting (high quality if all pre‐specified outcomes were reported such that they could be entered in an summary data meta‐analysis). In addition, we included ratings of adherence (high quality if ≥80% of patients had high attendance, defined as ≥80% of sessions attended) and contamination (high quality if no or limited adoption (<20%) of the intervention in the control group) as other potential sources of bias. Items related to blinding were omitted because blinding of patients and personnel is difficult in case of a PSI. Also, the rating of blinding of outcome assessors was excluded because QoL, EF, and SF were assessed using patient‐reported outcomes (PROs). Quality assessment of both reviewers were compared, and disagreements were resolved by discussion and consulting a third researcher (LB).

### 2.4 Outcome variables

QoL, EF, and SF were assessed with PROs (Table S2). In the present paper, we used baseline (pre‐intervention) and immediate or closest to post‐intervention values of the outcomes. Although we acknowledge the importance of long‐term intervention effects, this paper focuses on direct (short‐term) effects of the intervention, because follow‐up data was provided for only half of the studies which also used different follow‐up durations. To allow pooling of the different PROs, we recoded the individual scores into z‐scores by subtracting the mean score at baseline from the individual score, then dividing the result by the mean standard deviation at baseline. Subsequently, the pooled z‐scores were used for further analyses. If studies used both a cancer‐specific and a generic QoL PRO, data from the cancer‐specific PRO were used.

### 2.5 Possible moderators

The potential moderators tested in this IPD meta‐analysis were identified from previous original RCTs or meta‐analyses.^1^, ^2^, ^6^, ^7^, ^16^, ^19^, ^32^, ^33^ Potential demographic moderators included age, sex, marital status, and education level. We dichotomized marital status into single and/or living alone versus married and/or living with partner. As a consequence of different coding schemes used in the original RCTs, education level was dichotomized into low‐medium (primary or secondary school, and lower or secondary vocational education) or high (higher vocational, college, or university education).

Potential clinical moderators included type of cancer, type of treatment, and the presence of distant metastases. The type of cancer was categorized into breast, male genitourinary, gastrointestinal, hematological, gynecological, respiratory tract, and other types. We also checked moderator effects of breast cancer versus other types of cancer. Treatment with surgery, chemotherapy, radiotherapy, or hormone therapy were each dichotomised into previous or current treatment versus no such treatment.

Personal moderators included baseline values of QoL, EF, and SF (z‐scores).

Intervention type was categorized into information, support, CST, or psychotherapy, according to the classification model of Cunningham et al.^27^ Timing of intervention delivery was categorized into pre‐ anti‐cancer treatment, during treatment, post‐treatment, and end‐of‐life.^34^ As studies on interventions delivering PSI pre‐treatment and during end‐of‐life were not available, and only 1 study delivered PSI both pre‐and post‐treatment, we tested differences in intervention effects between those delivered during and post‐treatment. As hormone therapy for breast cancer may continue for several years post‐treatment, we considered women on hormone therapy who completed other primary cancer treatments as being post‐treatment. Men receiving androgen deprivation therapy for prostate cancer were considered as being during treatment. Intervention duration was dichotomized based on the median (≤12 weeks; >12 weeks). Interventions targeting patients with distress (eg, depression, fatigue, cognitive problems, symptoms) were dichotomized into yes or no.

### 2.6 Statistical analysis

We conducted 1‐step IPD meta‐analyses to study the effects and moderators of PSI on QoL, EF, and SF. The effects were evaluated by regressing the post‐intervention value (z‐score) of the outcome onto the intervention using linear mixed model analyses with a 2‐level structure (patients as level one and study as level 2) to take into account the clustering of patients within studies by using a random intercept on study level. The baseline value of the outcome (z‐score), age and cancer type were included in the model as covariates. The residuals of the models were distributed normally. Moderators of the intervention effects were examined by adding the moderator and its interaction term with the intervention into the regression model, for each moderator separately. To reduce ecological bias for patient‐level interactions, we separated within‐trial interaction from between‐trial interaction by centering the individual value of the covariate around the mean study value of that covariate.^24^ In case a RCT had 3 study arms with different study‐level moderators across study arms, interaction testing for a study‐level moderator was not possible. Therefore, in those situations, we tested differences between subgroups using dummy variables.

If the likelihood ratio test of the model with and without interaction term was significant (*P* < 0.05), strata were built, and the moderator analyses were repeated in the strata that included data from more than 1 RCT. Because type of intervention was the most significant moderator, we re‐examined the other potential moderators of intervention effects within the strata based on type of intervention (CST and psychotherapy). Because the majority of patients were women with breast cancer that followed CST, we performed a sensitivity analysis in this subgroup of patients.

Regression coefficients and 95% confidence intervals (CI) were reported, which represent the between group difference in z‐scores of QoL, EF, and SF, and correspond to a Cohen's d effect size. According to Cohen,^35^ d = 0.2 was considered small, d = 0.5 medium, and d = 0.8 large, respectively. The statistical analyses were conducted in SPSS 22.0 (IBM Corp. Released 2013. IBM SPSS Statistics for Windows, Version 22.0. Armonk, NY: IBM Corp.) and RStudio.^36^

## 3 RESULTS

### 3.1 Characteristics of studies and patients

**Figure 1:**
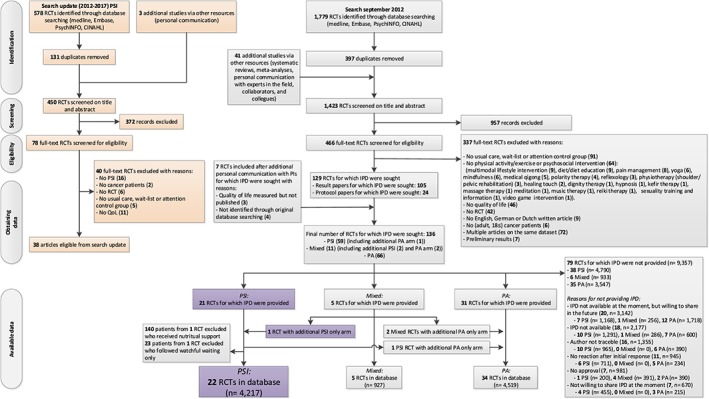
Flowchart of inclusion of randomized controlled trials (RCTs) in the POLARIS study. For this study on the effects and moderators of psychosocial interventions, individual patient data (IPD) of 22 RCTs were available. PA, physical activity interventions; PI, principal investigator; PSI, psychosocial interventions

Of the 136 RCTs that met the inclusion criteria for the POLARIS study in the original search, 59 RCTs evaluated the effects of PSI, and 2 RCTs^37^, ^38^ that evaluated the effects of physical activity combined with PSI also included a third study arm with PSI only (Figure 1). PIs of 22 of the 61 eligible RCTs (response 36%)^37^, ^39^, ^40^, ^41^, ^42^, ^43^, ^44^, ^45^, ^46^, ^47^, ^48^, ^49^, ^50^, ^51^, ^52^, ^53^, ^54^, ^55^, ^56^, ^57^, ^58^, ^59^ shared their data. In 1 RCT focusing on hematological cancer,^41^ we excluded patients who followed watchful waiting only (*n* = 23), as they did not fit into one of the intervention categories. In 1 RCT that included patients with mixed cancer types,^50^ we excluded patients with gastrointestinal cancer as they received PSI combined with nutritional support (*n* = 140). The final dataset included 4217 patients with cancer of whom 2215 were randomly allocated to the intervention and 2002 to the control group.

In total, 86% of the included RCTs reported random sequence generation, 73% reported adequate allocation concealment, 77% had adequate completeness of outcome data, 82% had complete outcome reporting, 41% described adequate intervention adherence, and 18% provided information on contamination (Table S1).

The mean age of participants was 56.0 (standard deviation = 11.4) years, 65% were female, 70% were married and/or lived with a partner, 33% were highly educated, 52% were diagnosed with breast cancer, and 9% had a distant metastatic disease at baseline (Table S2). Nineteen^37^, ^39^, ^40^, ^41^, ^42^, ^44^, ^45^, ^46^, ^47^, ^48^, ^49^, ^50^, ^52^, ^53^, ^54^, ^55^, ^56^, ^57^, ^59^ RCTs evaluated the effects of CST, two^43^, ^58^ evaluated the effects of psychotherapy, and one^51^ evaluated information only, 17 were conducted post‐cancer treatment, and 8 RCTs targeted patients with distress (Table S2).

### 3.2 Representativeness of included studies

The updated search yielded 38 additional RCTs. Of the 99 eligible RCTs, 50 reported summary data on QoL, 47 on EF, and 39 on SF. Of the 22 RCTs included in the IPD meta‐analyses, 10 published summary data on QoL, 13 on EF, and 8 on SF. We found no significant differences in effects on QoL (*P* = 0.10), EF (*P* = 0.47), and SF (*P* = 0.66) between RCTs of which IPD were shared (QoL: β = 0.10,95%CI = −0.03;0.24, EF: β = 0.13,95%CI = 0.02;0.25, SF: β = 0.12,95%CI = −0.03;0.27) and those of which IPD were not shared (QoL: β = 0.25,95%CI = 0.14;0.36, EF: β = 0.19,95%CI = 0.08;0.31, SF: β = 0.16,95%CI = 0.05;0.27) (Table S3).

The Eggers test was not statistically significant for all eligible and RCTs included reporting on QoL, EF, and SF, suggesting no evidence for publication bias.

### 3.3 Effects and moderators of PSI on QoL EF and SF

PSI significantly improved QoL (β = 0.14,95%CI = 0.06;0.21), EF (β = 0.13,95%CI = 0.05;0.20), and SF (β = 0.10,95%CI = 0.03;0.18), see Table 1 and Figure S1. Intervention effects on QoL (*P* = 0.05), EF (*P* < 0.01), and SF (*P* = 0.05) were significantly larger for younger patients. Intervention effects on EF (*P* = 0.03) were larger for patients who were single and/or living alone (β = 0.29,95%CI = 0.18;0.40) compared with married and/or living with partner (β = 0.09,95%CI = 0.03;0.15). Effects on EF differed by cancer type (*P* = 0.02). Effects on QoL (*P* = 0.01) and EF (*P* = 0.03) were larger for patients who were treated with chemotherapy. Intervention effects on EF were significantly larger for patients who did not receive radiotherapy (*P* = 0.05). Intervention effects on EF (*P* = 0.02) were larger for patients with lower EF at baseline. Type of PSI (*P* ≤ 0.01) significantly moderated the effects on QoL, EF, and SF, with largest effects for psychotherapy (QoL: β = 0.32,95%CI = 0.12;0.51, EF: β = 0.31,95%CI = 0.10;0.53, SF: β = 0.38,95%CI = 0.16;0.61). Intervention effects on QoL (*P* < 0.01), EF (*P* = 0.01), and SF (*P* < 0.01) were significantly larger in studies that specifically targeted patients with distress.

**Table 1:** Effects and moderators of psychosocial interventions on quality of life (QoL), emotional function, and social function. Regression coefficients (β) and 95% confidence intervals (CI) of the intervention effects, and *P*‐value of the likelihood ratio test of models with and without interactions are presented

	QoL		Emotional Function		Social Function	
	β (95% CI)	*P*	β (95% CI)	*P*	β (95% CI)	*P*
*Effect of psychosocial interventions*	0.14(0.06;0.21)*		0.13(0.05;0.20)*		0.10(0.03;0.18)*	
Age, years		0.05		<0.01		0.05
<50 years	0.25(0.15; 0.36)*		0.22(0.11;0.33)*		0.24(0.14;0.34)*	
50–70 years	0.08(0.01;0.14)*		0.11(0.05;0.17)*		0.06(−0.00;0.12)	
≥70 years	0.07(−0.06;0.20)		−0.01(−0.14;0.12)		0.03(−0.10;0.15)	
Sex (men vs women)		0.15		0.85		0.87
Marital status		0.55		0.03		0.88
Single/ living alone	…		0.29(0.18;0.40)*		…	
Married/ living with partner	…		0.09(0.03;0.15)*		…	
Education level (low‐medium vs high)		0.41		0.66		0.40
Type of cancer		0.35		0.02		0.89
Breast	…		0.15(0.08;0.23)*		…	
Genitourinary	…		0.07(−0.00;0.15)		…	
Hematological	…		0.14(−0.11;0.38)		…	
Gastrointestinal	…		−0.10(−0.36;0.16)		…	
Gynecological	…		0.27(−0.06;0.60)		…	
Lung	…		0.23(−0.06;0.51)		…	
Other	…		−0.66(−1.47;0.16)		…	
Type of cancer (breast vs other)		0.19		0.97		0.59
Distant metastasis at baseline		0.64		0.60		0.60
Surgery		0.81		0.40		0.08
Chemotherapy		0.01		0.03		0.14
No	0.03(−0.04;0.10)		0.06(−0.01;0.12)		…	
Yes	0.22(0.15;0.29)*		0.20(0.12;0.27)*		…	
Radiotherapy		0.80		0.05		0.09
No	…		0.16(0.08;0.23)*		…	
Yes	…		0.09(0.02;0.16)*		…	
Hormone therapy for breast cancer		0.88		0.61		0.06
Hormone therapy for prostate cancer		0.75		0.17		0.66
Baseline value of outcome a		0.40		0.02		0.14
<−0.5 SD	…		0.17(0.05;0.29)*		…	
−0.5 to 0.5 SD	…		0.14(0.06;0.23)*		…	
>0.5 SD	…		0.08(0.01;0.15)*		…	
Type of intervention		0.01		0.01		<0.01
Providing information	0.19(0.03;0.34)*		0.11(−0.06;0.28)		0.06(−0.09;0.22)	
Support	‐		‐		‐	
CST	0.09(0.04;0.15)*		0.10(0.04;0.15)*		0.08(0.03;0.13)*	
Psychotherapy	0.32(0.12;0.51)*		0.31(0.10;0.53)*		0.38(0.16;0.61)*	
Timing of intervention delivery (during vs post‐treatment)		0.81		0.31		0.69
Targeted intervention		<0.01		0.01		<0.01
No	0.07(0.02;0.12)*		0.09(0.04;0.14)*		0.06(0.01;0.11)*	
Yes	0.32(0.20;0.43)*		0.21(0.06;0.35)*		0.26(0.14;0.38)*	
Intervention duration (≤12 week vs >12 weeks)		0.14		0.27		0.26

Abbreviation: SD, standard deviation.

a Baseline QoL as moderator for outcome QoL, baseline emotional function as moderator for outcome emotional function, and baseline social function as moderator for outcome social function.

* *P* < 0.05.

### 3.4 Stratified analyses per intervention type

#### 3.4.1 Effects and moderators of coping skills training (19 RCTs)

CST significantly improved QoL (β = 0.11,95%CI = 0.03;0.20), EF (β = 0.10,95% CI = 0.02;0.18), and SF (β = 0.09,95%CI = 0.04;0.15), see Table 2. Patients who were younger had larger effects of CST on EF (*P* = 0.01) and SF (*P* = 0.03). Patients treated with chemotherapy had larger CST effects on QoL and EF (*P* = 0.01). Patients treated with surgery had larger effects on SF (*P* = 0.04). Effects on SF was also larger in women with breast cancer who did not receive hormone therapy (*P* = 0.01). Effects on QoL (*P* < 0.01) were larger in studies that targeted patients with distress. Sensitivity analyses among patients with breast cancer (*n* = 1753) showed larger CST effects on EF (*P* = 0.03) in patients treated with chemotherapy.

**Table 2:** Effects and moderators of coping skills training (CST) on quality of life (QoL), emotional function, and social function. Regression coefficients (β) and 95% confidence intervals (CI) of the intervention effects, and *P*‐value of the likelihood ratio test of models with and without interactions are presented

	QoL		Emotional Function		Social Function	
	β (95% CI)	*P*	β (95% CI)	*P*	β (95% CI)	*P*
*Effect of CST interventions*	0.11(0.03;0.20)*		0.10(0.02;0.18)*		0.09(0.04;0.15)*	
Age, years		0.11		0.01		0.03
<50 years	…		0.19(0.07;0.32)*		0.24(0.12;0.36)*	
50–70 years	…		0.09(0.02;0.16)*		0.04(−0.03;0.11)	
≥70 years	…		−0.02(−0.16;0.11)		0.03(−0.11;0.17)	
Sex (men vs women)		0.08		0.77		0.84
Marital status (single/living alone vs Married/living with partner)		0.33		0.06		0.68
Education level (low‐medium vs high)		0.74		0.79		0.57
Type of cancer		0.81		0.56		0.27
Type of cancer (breast vs other)		0.39		0.63		0.40
Distant metastasis at baseline		0.58		0.61		0.47
Surgery		0.75		0.53		0.04
No	…		…		−0.03(−0.15;0.09)	
Yes	…		…		0.14(0.07;0.20)*	
Chemotherapy		0.01		0.01		0.08
No	0.01(−0.06;0.08)		0.03(−0.04;0.10)		…	
Yes	0.21(0.13;0.29)*		0.18(0.09;0.27)*		…	
Radiotherapy		0.89		0.24		0.19
Hormone therapy for breast cancer		0.59		0.42		0.01
No	…		…		0.23(0.12;0.35)*	
Yes	…		…		0.05(−0.05;0.15)	
Hormone therapy for prostate cancer		0.85		0.17		0.63
Baseline value of outcome a		0.83		0.14		0.13
Timing of intervention delivery (during vs post‐treatment)		0.36		0.76		0.35
Targeted intervention		<0.01		0.34		0.18
No	0.06(0.00;0.12)*		…		…	
Yes	0.30(0.16;0.45)*		…		…	
Intervention duration (≤12 week vs >12 weeks)		0.16		0.27		0.26

Abbreviation: SD, standard deviation.

a Baseline QoL as moderator for outcome QoL, baseline emotional function as moderator for outcome emotional function, and baseline social function as moderator for outcome social function.

* *P* < 0.05.

#### 3.4.2 Effects and moderators of psychotherapy (2 RCTs)

Psychotherapy significantly improved QoL (β = 0.45,95%CI = 0.15;0.75), EF (β = 0.36,95%CI = 0.06;0.66), and SF (β = 0.34,95%CI = 0.07;0.62), see Table 3. Type of cancer moderated the intervention effects of psychotherapy on EF (*P* = 0.02). Intervention effects on EF were significant for patients with breast (β = 0.46,95%CI = 0.06;0.87) and hematological cancer (β = 1.11,95%CI = 0.34;1.87).

**Table 3:** Effects and moderators of psychotherapy interventions on quality of life (QoL), emotional function, and social function. Regression coefficients (β) and 95% confidence intervals (CI) of the intervention effects, and *P*‐value of the likelihood ratio test of models with and without interactions are presented

	QoL		Emotional Function		Social Function	
	β (95% CI)	*P*	β (95% CI)	*P*	β (95% CI)	*P*
*Effect of psychotherapy*	0.45(0.15;0.75)*		0.36(0.06;0.66)*		0.34(0.07;0.62)*	
Age, years		0.50		0.22		0.58
Sex (men vs women)		0.54		0.62		0.34
Marital status (single/living alone vs married/living with partner)		0.68		0.25		0.56
Education level (low‐medium vs high)		0.22		0.14		0.74
Type of cancer		0.07		0.02		0.38
Breast	…		0.46(0.06;0.87)*		…	
Genitourinary	…		0.49(−0.04;1.03)		…	
Hematological	…		1.11 (0.34;1.87)*		…	
Gastrointestinal	…		−0.70(−1.65;0.24)		…	
Gynecological	…		0.36(−0.02;0.75)		…	
Lung	…		‐		…	
Other	…		−0.86(−2.72;1.01)		…	
Type of cancer (breast vs other)		0.22		0.49		1.00
Surgery		0.31		0.23		0.19
Chemotherapy		0.64		0.66		0.30
Radiotherapy		0.08		0.09		0.09
Hormone therapy for breast cancer		0.51		0.38		0.78
Baseline value of outcome a		0.74		0.20		0.49
Timing of intervention delivery (during vs post‐treatment)		0.31		0.23		0.24

Abbreviation: SD, standard deviation.

a Baseline QoL as moderator for outcome QoL, baseline emotional function as moderator for outcome emotional function, and baseline social function as moderator for outcome social function.

* *P* < 0.05.

## 4 DISCUSSION

This IPD meta‐analysis of 22 RCTs, including 4217 patients with cancer, showed that PSI significantly improved QoL, EF, and SF, with small overall effects, both during and after treatment. The present IPD meta‐analysis enabled the testing of potential moderators of intervention effects using interaction tests in a large sample. In the current sample, of which half of the population was diagnosed with breast cancer and one third with genitourinary cancer, we found significant differences in effects of different types of PSI, with largest effects of psychotherapy in comparison with CST and providing information. The effects of CST were moderated by age, treatment type, and by targeted interventions. The effects of psychotherapy on EF may be moderated by cancer type, but these analyses were based on 2 RCTs with small sample sizes of some cancer types.

Our finding that the effects on QoL, EF, and SF were larger for psychotherapy than for CST differs from a previous summary data meta‐analysis that summarized the results of 37 RCTs in a mixed cancer population and reported no difference in effects between information provision (6 RCTs), support (4 RCTs), CST (20 RCTs), and psychotherapy (7 RCTs).^12^ However, our finding should be interpreted with caution, because we were only able to include 2 RCTs evaluating psychotherapy interventions, and they were offered to patients with mixed cancer types^43^ or metastatic breast cancer.^58^ These 2 RCTs also targeted patients with higher levels of depressive symptoms, which may explain the larger effects of psychotherapy compared with CST.^60^

The larger effects of CST in younger patients found in the current IPD meta‐analysis may be explained by the higher psychological distress and supportive care needs of younger patients in physical, informational, and emotional domains.^61^, ^62^ Consequently, CST may more effectively improve EF and SF for this subgroup of patients. Alternatively, older patients with cancer may have specific needs that were not, or only partly, addressed by CST.^61^ There is limited knowledge, however, about the supportive care needs of elderly patients with cancer, who more often have comorbid conditions.^61^ Further research is needed to identify the supportive care needs of elderly patients with cancer and to develop effective CST targeting this population.

Treatment type was a significant moderator effect of CST, such that larger effects on QoL and EF were found in patients treated with chemotherapy, and effects on SF were larger in patients with breast cancer that did not receive hormone therapy, and in patients who had surgery. The larger effects of CST in patients treated with chemotherapy compared with those who were not may be explained by the specific side effects of chemotherapy, including fatigue,^63^ pain,^64^ and emotional or cognitive problems,^65^ which are specifically targeted by CST. The larger effects in patients who did not receive hormone therapy may also be caused by milder side effects of hormone therapy, compared with chemotherapy. Additionally, patients with hormone‐sensitive tumors generally have a lower risk of disease recurrence than patients with hormone‐insensitive tumors.^66^ The larger effects of CST on SF in patients who had surgery should be interpreted with caution as this may vary by type of surgery (eg, radical mastectomy versus breast‐preserving surgery^67^). Additionally, we used broad categories of treatment in this heterogeneous group of patients and treatment combinations and intervention timing may vary. Future studies should therefore examine moderator effects of cancer treatment within more homogeneous groups of patients. Our sensitivity analyses in women with breast cancer showed larger CST effects on EF in those treated with chemotherapy, emphasizing that CST is particularly beneficial in women with breast cancer treated with chemotherapy.

We observed a larger effect of CST on QoL in RCTs that specifically targeted patients with higher levels of distress before the intervention. This underlines the importance of targeting patients with distress so that the limited available resources for CST can be targeted to those who need and benefit most from CST. Unexpectedly, despite larger effects in targeted studies, no moderator effect of the baseline value of QoL, EF, and SF was found. Also, previous studies on the moderator effect of baseline distress were inconsistent.^1^, ^5^, ^18^, ^60^, ^68^

In the 2 RCTs that studied the effects of psychotherapy, that specifically targeted patients with distress, we found a significant moderator effect of cancer type. Effects on EF were significant for patients with breast and hematological cancer. Due to the small sample size of some cancer types, future studies should confirm whether patients with different cancer types indeed respond differently to interventions.

### 4.1 Strengths and limitations

Strengths of this study include the IPD approach and the large number of RCTs from multiple countries and the resulting large sample size that enabled testing of interactions between the intervention and patient‐level characteristics and conducting subsequent stratified analyses, as well as the uniform analytical procedures across all studies. The study also had a number of limitations that should be noted. First, the pooled RCTs were heterogeneous with respect to type of intervention and cancer. Future studies with more homogeneous patient samples are needed to investigate potential moderator effects of PSI‐related characteristics and techniques such as delivery format (eg, individual, group, or couple therapy), method (eg, face‐to‐face, telephone, or web‐based), and profession (eg, psychologist versus nurse). Also, other potential psychosocial moderators of PSI effects such as coping skills, self‐esteem, and perceived social support were not explored,^19^, ^69^ and should therefore be examined in future studies. Another limitation is the time between the literature search and the current publication. The collection of IPD from multiple RCTs is very time consuming, and it took more than 3 years to collect these data, which is comparable to IPD meta‐analysis in other fields of research.^22^ In addition, during these 3 years, we maintained contact with PIs of ongoing studies (*n* = 6) of which protocol papers were identified, and these were included in the current IPD meta‐analysis. The results of the moderator analyses, however, are novel and valid. Third, only 36% of the eligible RCTs were included in the IPD meta‐analysis, which may limit the generalizability of the results.^70^ However, we found no differences in effect sizes between RCTs included and those not included, indicating that the 22 RCTs included in the analyses were a representative sample of the published studies. Additionally, the results of the current analyses depend on the studies conducted so far, thus mainly among patients with breast and genitourinary cancer, and may therefore not be generalizable to other cancer populations. Fourth, some biases were present in the included RCTs, with little information on adherence to the PSI and potential contamination in the control group. Adherence and contamination may influence the intervention effect as well. With study quality being a study‐level characteristic of which the power is determined by the number of studies, it is difficult to disentangle the impact of study quality versus other intervention‐related characteristics and techniques on the moderator effects. Therefore, the quality rating was added to inform the reader about the overall study quality. Finally, as 11 of the 22 RCTs did not provide sufficient data at follow‐up or used different follow‐up durations, we were not able to study the intervention effects at long‐terms.

### 4.2 Clinical implications

Our study showed that PSI significantly improves QoL, EF, and SF both during and post cancer treatment, but the overall effects are small. Psychotherapy appears to have larger effects compared with CST, but this conclusion is based on just 2 psychotherapy interventions that specifically targeted patients with distress. The effects of existing CST were larger for interventions that were targeted, and in patients who were younger. Additionally, treatment type moderated the effects of CST. CST was particularly beneficial in patients treated with chemotherapy. Our study highlights the importance of targeted interventions, and it presents the need of developing interventions tailored to the specific needs of elderly patients.

## CONFLICT OF INTEREST

Dr Chambers reports personal fees from Tolmar (speakers' bureau); Dr Horne reports to be director and shareholder (Pharmed Research Ltd) and a UCL business spin out company (Spoonful of Sugar Ltd), providing consultancy on medication‐related behaviors to health care policy makers, providers, and industry.

## Supporting information


**Figure S1.** Forest plots of the effects of psychosocial interventions on quality of life, emotional function, and social function

**Table S1.** Characteristics of the 22 included randomized controlled trials on the effect of psychosocial interventions, in alphabetical order of first author.

**Table S2.** Demographic, clinical, personal and intervention‐related characteristics, quality of life, emotional function, and social function of patients in the intervention and control group.

**Table S3.** Representativeness and publication bias of the pooled effects of studies providing data for the POLARIS study and those not providing data.
