# Supplementary material for: Effects and moderators of psychosocial interventions on quality of life, and emotional and social function in patients with cancer: An individual patient data meta‐analysis of 22 RCTs
Source: Psychooncology. 2018 Mar 15;27(4):1150–61. doi: 10.1002/pon.4648 (PMC5947559; doi:10.1002/pon.4648)
Supplement: Supplementary file 1 — Figure S1. Forest plots of the effects of psychosocial interventions on quality of life, emotional function, and social function [file PON-27-1150-s001.docx]

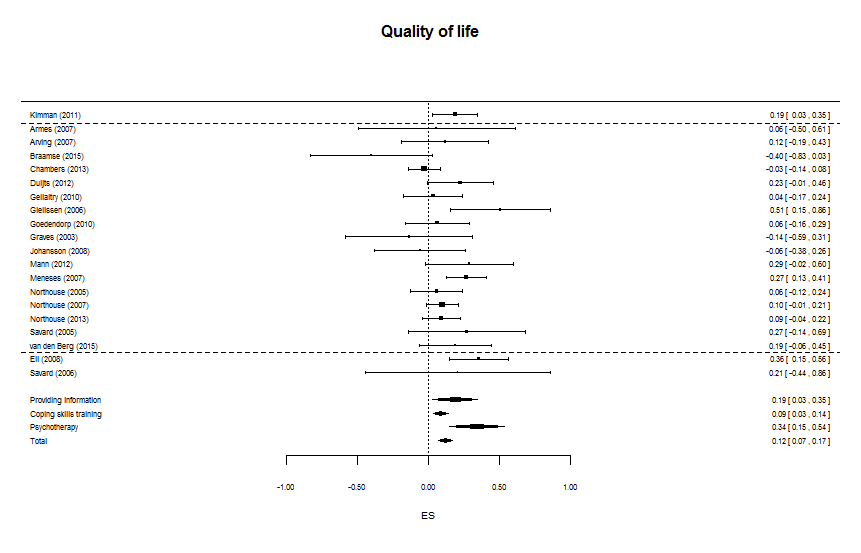

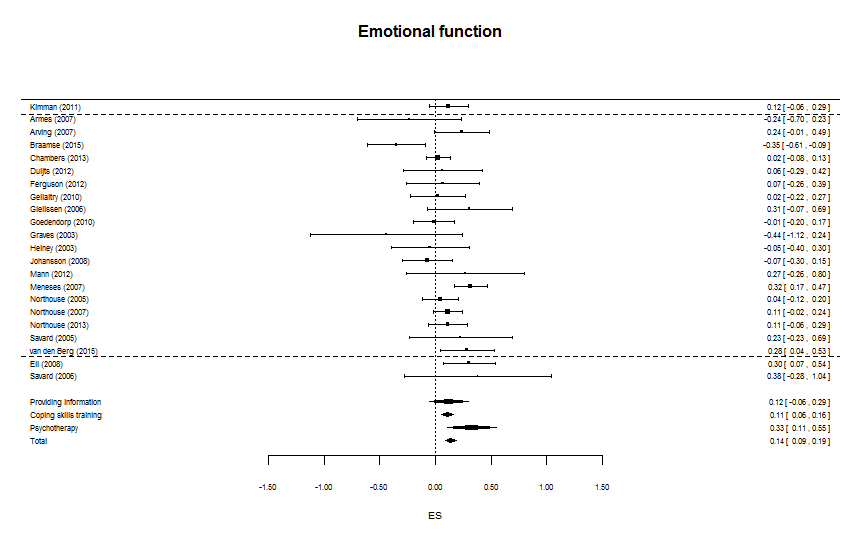

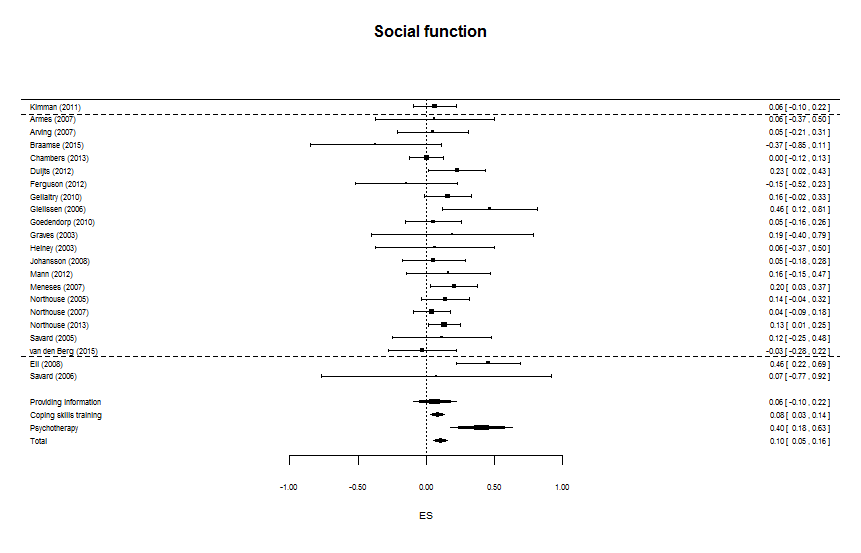


**FIGURE LEGEND**

Supplemental Figure 1. Forest plots of the effects of psychosocial interventions on quality of life, emotional function, and social function

Data represent the regression coefficients [95% confidence intervals] of the effects of psychosocial intervention on quality of life, emotional function, and social function (in z-scores). The intervention *providing information* is presented above the first dashed line, *coping skills training* interventions between the first and second dashed line and *psychotherapy* interventions below the second dashed line.
