# Supplementary material for: Effects and moderators of psychosocial interventions on quality of life, and emotional and social function in patients with cancer: An individual patient data meta‐analysis of 22 RCTs
Source: Psychooncology. 2018 Mar 15;27(4):1150–61. doi: 10.1002/pon.4648 (PMC5947559; doi:10.1002/pon.4648)
Supplement: Supplementary file 2 — Table S1. Characteristics of the 22 included randomized controlled trials on the effect of psychosocial interventions, in alphabetical order of first author. [file PON-27-1150-s002.docx]

Supplemental Table S1. Characteristics of the 22 included randomized controlled trials on the effect of psychosocial interventions, in alphabetical order of first author.

|  |  |  |  |  |  |  |  | **Intervention** | | | | | | | **Control** | **Quality** | | | | | | |
| --- | --- | --- | --- | --- | --- | --- | --- | --- | --- | --- | --- | --- | --- | --- | --- | --- | --- | --- | --- | --- | --- | --- |
| **Author**  **(year)** | **Country** | **Sample (n)** | **Sex**  **(% male)** | **Age, mean** | **Diagnosis** | **Timing** | **Targeted** | | **Type** | **Format** | **Method** | **Duration**  **(weeks)** | **Sessions** | **Profession** |  | **PRO**  **(P or S)** | **RSG** | **AC** | **IO** | **IR** | **Adh** | **Con** |
| Armes,  2007 | UK | 55 | 40 | 59 | Mixed | During CT | Yes | | CST | Individual | FTF | 12 | 3 | Nurse | UC | QLQ-C30  S | + | + | + | + | - | ? |
| Arving,  2007 | SWE | 179 | 0 | 55 | Breast | During | No | | CST | Individual | FTF | 4 | NR | Nurse or psychologist | UC | QLQ-C30  P | + | ? | + | + | ? | ? |
| Braamse,  2015 | NL | 72 | 72 | 54 | Hematological | Post high-dose CT and auto-SCT | No | | CST | Individual | Web | 13 | NR | Psychologist | UC | QLQ-C30  S | + | + | + | + | - | + |
| Chambers,  2013 | AUS | 740 | 100 | 61 | Prostate | Pre and post | No | | CST | Individual | Tel | 8 | 5 | Nurse | UC | SF-36  P | + | + | + | + | + | ? |
| Duijts,  2012 | NL | 212 | 0 | 48 | Breast | Post CT and/or HT | Yes | | CST | Group | FTF | 12 | 6 | Psychologist | WLC | SF-36  S | + | + | + | + | - | ? |
| Ell,  2008 | USA | 472 | 16 | 49 | Mixed | During or post | Yes | | PT | Individual | FTF | 52 | 0 – 54 | Cancer depression clinical specialists | AC | FACT-G  P | + | + | + | + | - | ? |
| Ferguson,  2012 | USA | 40 | 0 | 50 | Breast | Post CT | Yes | | CST | Individual | FTF | 8 | 4 | Psychologist | WLC | QOL-CS  P | + | + | + | + | ? | ? |
| Gellaitry,  2010 | UK | 93 | 0 | 58 | Breast | Post RT | No | | CST | Individual | Tel | 4 | 4 | Researcher | UC | FACT-G  P | + | ? | - | - | ? | ? |
| Gielissen,  2006 | NL | 98 | 51 | 45 | Mixed | Post | Yes | | CST | Individual | FTF | 26 | 5 – 26 | Psychologist | WLC | QLQ-C30  S | ? | + | + | + | ? | ? |
| Goedendorp, 2010 | NL | 148 | 36 | 56 | Mixed | During | No | | CST | Individual | FTF | 26 | 1-10 | Psychologist | UC | QLQ-C30  S | + | + | + | - | ? | ? |
| Graves,  2003 | USA | 32 | 0 | 56 | Breast | During or post | No | | CST | Group | FTF | 8 | 8 | Psychologist | UC | FACT-G  P | ? | ? | - | - | ? | ? |
| Heiney,  2003 | USA | 66 | 0 | 50 | Breast | Post | No | | CST | Group | Tel | 6 | 6 | Group therapist | UC | QOL-CS  P | + | ? | - | + | + | ? |
| Johansson,  2008 | SWE | 171 | 35 | 62 | Breast &  Prostate | During | No | | CST | Individual | FTF | 12 | 1 – 24 | Psychologist | UC | QLQ-C30  P | + | + | ? | + | ? | ? |
| Kimman,  2011 | NL | 299 | 0 | 56 | Breast | Post | No | | Info | Group | FTF | 12 | 2 | Psychologist | UC | QLQ-C30  P | + | + | + | + | + | ? |
| Mann,  2012 | UK | 96 | 0 | 53 | Breast | Post | Yes | | CST | Group | FTF | 9 | 6 | Psychologist | UC | SF-36  S | + | + | + | + | + | ? |
| Meneses,  2007 | USA | 261 | 0 | 55 | Breast | Post | No | | CST | Individual | FTF | 12 | 3 | Nurse | WLC | QOL-CS  P | ? | ? | + | - | ? | ? |

**Supplemental Table S1 (continued)**

| **Author (year)** | **Country** | **Sample** | **Sex**  **(% male)** | **Age, mean** | **Diagnosis** | **Timing** | **Targeted** | **Type** | **Format** | **Method** | **Duration**  **(weeks)** | **Sessions** | **Profession** |  | **Q**  **(P or S)** | **RSG** | **AC** | **IO** | **IR** | **Adh** | **Con** |
| --- | --- | --- | --- | --- | --- | --- | --- | --- | --- | --- | --- | --- | --- | --- | --- | --- | --- | --- | --- | --- | --- |
| Northouse,  2005 | USA | 192 | 0 | 54 | Recurrent breast | During or post | No | CST | Couple | FTF | 12 | 5 | Nurse | UC | FACT-G  P | + | + | + | + | + | + |
| Northouse,  2007 | USA | 263 | 100 | 63 | Prostate | During or post | No | CST | Couple | FTF | 16 | 5 | Nurse | UC | FACT-G  P | + | + | + | + | + | + |
| Northouse,  2013 | USA | 484 | 38 | 60 | Advanced lung, colorectal, breast, and prostate | During or post | No | CST | Couple | FTF | 12 | 3 (brief); 6 (ext.)^a^ | Nurse | UC | FACT-G  P | + | + | + | + | + | + |
| Savard,  2005 | CAN | 57 | 0 | 54 | Breast | Post CT and/or RT | Yes | CST | Group | FTF | 8 | 8 | Psychologist | WLC | QLQ-C30  P | + | ? | + | + | + | ? |
| Savard,  2006 | CAN | 37 | 0 | 51 | Metastatic  Breast | During or post | Yes | PT | Individual | FTF | 8 | 8 | Psychologist | WLC | QLQ-C30  P | + | + | - | + | ? | ? |
| van den Berg,  2015 | NL | 150 | 0 | 51 | Breast | Post | No | CST | Individual | Web | 16 | NR | None | UC | QLQ-C30  S | + | + | + | + | + | ? |

^a^ Patients followed a brief (brief) or extended (ext.) intervention.
AC=attention control group; AUS=Australia; CAN=Canada; CST=coping skills training; CT=chemotherapy; FACT-G=Functional Assessment of Cancer Therapy-General; FTF=face-to-face; HT=hormone therapy; NL=the Netherlands; NR=not reported; P=quality of life as primary outcome measure; PRO=patient reported outcome PT=psychotherapy; QLQ-C30=European Organization for Research and Treatment of Cancer Quality of Life Questionnaire – Core 30; QOL-CS=cancer-specific quality of life – cancer survivors; RT=radiotherapy; S=quality of life as secondary outcome measure; SF-36=Short Form-36 Item Health Survey; SWE=Sweden; UC=usual care; UK=United Kingdom; USA=United States of America; WLC=wait list control group.
*Quality assessment*: +=high quality; -=low quality; ?=unclear quality; RSG=random sequence generation; AC=allocation concealment; IO=incomplete outcome; IR=incomplete reporting; Adh=adherence; Con=contamination.
