# Supplementary material for: Effects and moderators of psychosocial interventions on quality of life, and emotional and social function in patients with cancer: An individual patient data meta‐analysis of 22 RCTs
Source: Psychooncology. 2018 Mar 15;27(4):1150–61. doi: 10.1002/pon.4648 (PMC5947559; doi:10.1002/pon.4648)
Supplement: Supplementary file 3 — Table S2. Demographic, clinical, personal and intervention‐related characteristics, quality of life, emotional function, and social function of patients in the intervention and control group. [file PON-27-1150-s003.docx]

**Supplemental Table S2.** Demographic, clinical, personal and intervention-related characteristics, quality of life, emotional function and social function of patients in the intervention and control group.

| **Variable** | **Intervention (n=2215)** | | **Control (n=2002)** | |
| --- | --- | --- | --- | --- |
| *Demographic* |  |  |  |  |
| Age, mean (SD) years | 56.1 (11.5) |  | 56.0 (11.2) |  |
| Age categories, n (%) |  |  |  |  |
| <50 years | 598 (27.0) |  | 553 (27.6) |  |
| 50–70 years | 1324 (59.8) |  | 1220 (60.9) |  |
| ≥70 years | 292 (13.2) |  | 227 (11.3) |  |
| Unknown | 1 (0.0) |  | 2 (0.1) |  |
| Sex, n (%) |  |  |  |  |
| Male | 773 (34.9) |  | 723 (36.1) |  |
| Female | 1442 (65.1) |  | 1279 (63.9) |  |
| Marital status, n (%) |  |  |  |  |
| Single/living alone | 555 (25.1) |  | 511 (25.5) |  |
| Married/living together | 1558 (70.3) |  | 1385 (69.2) |  |
| Unknown | 102 (4.6) |  | 106 (5.3) |  |
| Educational level, n (%) |  |  |  |  |
| Low/medium | 1130 (51.0) |  | 1031 (51.5) |  |
| High | 726 (32.8) |  | 678 (33.9) |  |
| Unknown | 359 (16.2) |  | 293 (14.6) |  |
| *Clinical* |  |  |  |  |
| Type of cancer, n (%) |  |  |  |  |
| Breast | 1153 (52.1) |  | 1039 (51.9) |  |
| Genitourinary | 625 (28.2) |  | 610 (30.5) |  |
| Gynecological | 117 (5.3) |  | 106 (5.3) |  |
| Gastrointestinal | 137 (6.2) |  | 91 (4.5) |  |
| Lung | 102 (4.6) |  | 61 (3.0) |  |
| Hematological | 64 (2.9) |  | 76 (3.8) |  |
| Other | 15 (0.7) |  | 17 (0.8) |  |
| Unknown | 2 (0.1) |  | 2 (0.1) |  |
| Distant metastasis at baseline, n (%) ^a^ |  |  |  |  |
| No | 1715 (77.4) |  | 1539 (76.9) |  |
| Yes | 196 (8.8) |  | 168 (8.4) |  |
| Unknown | 304 (13.7) |  | 295 (14.7) |  |
| Surgery, n (%) ^b^ |  |  |  |  |
| No | 441 (20.1) |  | 351 (18.0) |  |
| Prior to intervention | 1470 (67.1) |  | 1311 (67.1) |  |
| During intervention | 75 (3.4) |  | 67 (3.4) |  |
| Mid-intervention | 167 (7.6) |  | 189 (9.7) |  |
| Unknown | 38 (1.7) |  | 36 (1.8) |  |
| Chemotherapy, n (%) |  |  |  |  |
| No | 1058 (47.8) |  | 978 (48.9) |  |
| Prior to intervention | 579 (26.1) |  | 617 (30.8) |  |
| During intervention | 526 (23.7) |  | 357 (17.8) |  |
| Mid-intervention | 4 (0.2) |  | 2 (0.1) |  |
| Unknown | 48 (2.2) |  | 48 (2.4) |  |

**Supplemental Table S2 (continued)**

| **Variable** | **Intervention (n=2215)** | | **Control (n=2002)** | |
| --- | --- | --- | --- | --- |
| Radiotherapy, n (%) |  |  |  |  |
| No | 1023 (46.2) |  | 896 (44.8) |  |
| Prior to intervention | 647 (29.2) |  | 651 (32.5) |  |
| During intervention | 324 (14.6) |  | 226 (11.3) |  |
| Mid-intervention | 154 (7.0) |  | 160 (8.0) |  |
| Unknown | 67 (3.0) |  | 69 (3.4) |  |
| Hormone therapy |  |  |  |  |
| Breast cancer patients (n=2192), n (%) |  |  |  |  |
| No | 541 (46.9) |  | 445 (42.8) |  |
| Yes | 522 (45.3) |  | 503 (48.4) |  |
| Unknown | 90 (7.8) |  | 91 (8.8) |  |
| Prostate cancer patients (n=1159), n (%) |  |  |  |  |
| No | 371 (63.1) |  | 360 (63.0) |  |
| Prior to intervention | 5 (0.9) |  | 5 (0.9) |  |
| During intervention | 82 (13.9) |  | 83 (14.5) |  |
| Mid-intervention | 115 (19.6) |  | 115 (20.1) |  |
| Unknown | 15 (2.6) |  | 8 (1.4) |  |
| SCT, n (%) ^c^ |  |  |  |  |
| Allogenic SCT | 0 (0.0) |  | 0 (0.0) |  |
| Autologous SCT | 24 (37.5) |  | 48 (63.2) |  |
| Unknown | 40 (62.5) |  | 28 (36.8) |  |
| *Intervention-related ^d^* |  |  |  |  |
| Type of intervention, n (%) |  |  |  |  |
| Information only (k=1) | 149 (6.7) |  |  |  |
| Support (k=0) | 0 (0.0) |  |  |  |
| Coping skills training (k=19) | 1803 (81.4) |  |  |  |
| Psychotherapy (k=2) | 263 (11.9) |  |  |  |
| Timing intervention, n (%) ^e^ |  |  |  |  |
| Pre and post-treatment (k=1) | 372 (16.8) |  |  |  |
| During treatment (k=10) | 857 (38.7) |  |  |  |
| Post-treatment (k=17) | 986 (44.5) |  |  |  |
| Targeted intervention, n (%) |  |  |  |  |
| No (k=14) | 1672 (75.5) |  |  |  |
| Yes (k=8) | 543 (24.5) |  |  |  |
| Format intervention, n (%) |  |  |  |  |
| Individual therapy (k=13) | 1287 (58.1) |  |  |  |
| Group therapy (k=6) | 380 (17.2) |  |  |  |
| Couple therapy (k=3) | 548 (24.7) |  |  |  |
| Method delivery, n (%) |  |  |  |  |
| Face-to-face (k=17) | 1671 (75.4) |  |  |  |
| Telephone (k=3) | 450 (20.3) |  |  |  |
| Web-based (k=2) | 94 (4.2) |  |  |  |
| Profession conducting intervention, n (%) |  |  |  |  |
| Psychologist (k=10) | 664 (30.0) |  |  |  |
| Nurse (k=7) | 1137 (51.3) |  |  |  |
| Other (k=5) | 414 (18.7) |  |  |  |
| Type of control, n (%) ^f^ |  |  |  |  |
| Usual care (k=14) |  |  | 1374 (68.6) |  |
| Wait list control (k=6) |  |  | 350 (17.5) |  |
| Attention control (k=2) |  |  | 278 (13.9) |  |

**Supplemental Table S2 (continued)**

| **Variable** | **Intervention (n=2215)** | | **Control (n=2002)** | |
| --- | --- | --- | --- | --- |
|  | pre  Mean(SD) | post  Mean(SD) | pre  Mean(SD) | post  Mean(SD) |
| Quality of life, mean (SD) ^g^ |  |  |  |  |
| FACT-G, total score | 74.2 (18.8) | 79.3 (16.4) | 75.0 (18.1) | 77.0 (17.5) |
| EORTC QLQ-C30, subscale global QoL | 65.8 (20.6) | 71.3 (20.6) | 66.4 (20.1) | 69.4 (18.8) |
| QoL-CS, total score | 6.8 (1.4) | 7.2 (1.3) | 6.8 (1.5) | 6.9 (1.5) |
| SF-36, subscale general health | 69.0 (19.3) | 70.6 (19.0) | 69.6 (19.2) | 70.1 (20.0) |
| Emotional function, mean (SD) ^g^ |  |  |  |  |
| FACT-G, subscale EWB | 15.7 (4.9) | 17.4 (4.4) | 15.7 (4.6) | 16.6 (4.2) |
| EORTC QLQ-C30, subscale EF | 73.6 (22.0) | 80.2 (20.1) | 74.1 (21.5) | 78.0 (20.9) |
| QoL-CS, subscale PWB | 5.9 (1.7) | 6.3 (1.6) | 6.2 (1.7) | 6.1 (1.8) |
| SF-36, subscale EF | 80.7 (29.2) | 81.4 (27.8) | 83.5 (27.7) | 81.0 (27.6) |
| Social function, mean (SD) ^g^ |  |  |  |  |
| FACT-G, subscale SWB | 20.2 (6.2) | 21.2 (5.6) | 19.9 (5.9) | 19.6 (6.1) |
| EORTC QLQ-C30, subscale SF | 77.6 (25.0) | 83.9 (22.4) | 76.5 (25.8) | 82.5 (22.8) |
| QoL-CS, subscale SWB | 6.4 (1.7) | 7.1 (1.9) | 6.6 (1.8) | 7.0 (1.9) |
| SF-36, subscale SF | 82.2 (22.7) | 80.1 (23.2) | 85.0 (20.7) | 80.1 (23.3) |

EF=emotional function; EORTC QLQ-C30=European Organisation Research and Treatment of Cancer Quality of life questionnaire-Core 30; EWB=emotional well-being; FACT-G=Functional Assessment of Cancer Therapy-General; k=number of trials; n=number of patients; PWB=psychological well-being; QoL-CS=quality of life-cancer survivors; SF-36=Short Form-36 Health survey; SCT=stem cell transplantation; SD=standard deviation; SF=social function; SWB=social well-being.
^a^ proportion of patients of solid tumours (n=4145); ^b^ proportion of patients without SCT (n=4145); ^c^ proportion of patients with SCT (n=72); ^d^ proportion of patients from intervention groups (n=2215); ^e^ some trials included patients during and post-treatment (k=6) and therefore the total number of trials exceeds 22; ^f^ proportion of patients from the control groups (n=2002). ^g^ Higher scores represents higher QoL for FACT-G, EORTC QLQ-C30, QoL-CS, and SF-36.
