# Supplementary material for: Effects and moderators of psychosocial interventions on quality of life, and emotional and social function in patients with cancer: An individual patient data meta‐analysis of 22 RCTs
Source: Psychooncology. 2018 Mar 15;27(4):1150–61. doi: 10.1002/pon.4648 (PMC5947559; doi:10.1002/pon.4648)
Supplement: Supplementary file 4 — Table S3. Representativeness and publication bias of the pooled effects of studies providing data for the POLARIS study and those not providing data. [file PON-27-1150-s004.docx]

**Supplemental Table S3.** Representativeness and publication bias of the pooled effects of studies providing data for the POLARIS study and those not providing data.

|  |  | **Pooled effect** | **Test of heterogeneity** | | | **Between group differences** |
| --- | --- | --- | --- | --- | --- | --- |
| **Representativeness** | **k** | **g (95% CI)** | **Q** | **I^2^** | **p** | **p** |
| *Quality of life* |  |  |  |  |  |  |
| All eligible RCTs | 50 | 0.21 (0.12; 0.30)^*^ | 133.27 | 60.23 | <0.01 |  |
| RCTs providing data | 10 | 0.10 (-0.03; 0.24) | 16.92 | 40.91 | 0.08 |  |
| RCTs not providing data | 40 | 0.25 (0.14; 0.36)^*^ | 112.34 | 62.61 | <0.01 | 0.10 |
| *Emotional function* |  |  |  |  |  |  |
| All eligible RCTs | 47 | 0.17 (0.09; 0.26)^*^ | 135.21 | 61.54 | <0.01 |  |
| RCTs providing data | 13 | 0.13 (0.02; 0.25)^*^ | 25.79 | 45.71 | 0.03 |  |
| RCTs not providing data | 34 | 0.19 (0.08; 0.31)^*^ | 107.62 | 65.62 | <0.01 | 0.47 |
| *Social function* |  |  |  |  |  |  |
| All eligible RCTs | 39 | 0.14 (0.06; 0.23)^*^ | 75.04 | 46.69 | <0.01 |  |
| RCTs providing data | 8 | 0.12 (-0.03; 0.26) | 14.29 | 37.00 | 0.11 |  |
| RCTs not providing data | 31 | 0.16 (0.05; 0.27)^*^ | 60.65 | 50.53 | <0.01 | 0.66 |
| **Publication bias using trim and fill procedure** | **k_missing_** | **Adjusted effect** |  |  | **P_Egger_ ^a^** |  |
| *Quality of life* |  |  |  |  |  |  |
| All eligible RCTs | 0 | 0.21 (0.12; 0.30)^*^ |  |  | 0.21 |  |
| RCTs providing data | 0 | 0.10 (-0.03; 0.24) |  |  | 0.64 |  |
| *Emotional function* |  |  |  |  |  |  |
| All eligible RCTs | 0 | 0.17 (0.09; 0.26)^*^ |  |  | 0.42 |  |
| RCTs providing data | 0 | 0.13 (0.02; 0.24)^*^ |  |  | 0.69 |  |
| *Social function* |  |  |  |  |  |  |
| All eligible RCTs | 6 | 0.21 (0.11; 0.30)^*^ |  |  | 0.25 |  |
| RCTs providing data | 2 | 0.17 (0.01; 0.33)^*^ |  |  | 0.07 |  |

^a^ The Egger’s test investigates the publication bias captured by the funnel plot
k= number of trials; RCTs= randomized controlled trials
^*^p<0.05.
